# Retrospective Evaluation of Fascial Plane Blocks in Cardiac Surgery With Median Sternotomy in a Tertiary Hospital

**DOI:** 10.7759/cureus.35718

**Published:** 2023-03-03

**Authors:** Filiz Ata, Canan Yılmaz

**Affiliations:** 1 Anesthesiology and Reanimation, University of Bursa, Bursa Yüksek İhtisas Education and Research Hospital, Bursa, TUR

**Keywords:** analgesia, cardiac surgery, ultrasonography, fascial plane block, postoperative pain

## Abstract

Background and aim: Cardiac surgery typically causes moderate to severe postoperative pain and discomfort. Inadequate pain management in the early postoperative period leads to pulmonary complications. The length of intensive care unit (ICU) stay and the hospital is typically prolonged. As a component of multimodal analgesia regimens, fascial plane blocks have become more popular. In our clinic, serratus anterior plane blocks (SAPB), pectoral nerve blocks (PECS I-II), and pectointercostal nerve fascial plane blocks (PIFB) are performed by ultrasonography. We wished to evaluate the postoperative visual pain scale, initial additional analgesic agent requirement time, extubation time, morbidity and mortality in patients who underwent open heart surgery with fascial plane blocks.

Materials and methods: Forty-eight patients over 18 years who underwent open heart surgery with sternotomy between 01 September 2021 and 15 June 2022 were evaluated retrospectively. Only patients with chest wall blocks placed at the end of surgery were included in the study. In Group 1, the PECS II block was placed on the chest tube side and bilateral PIFBs were placed at the end of surgery in the operating room.

In Group 2, SAPB was placed on the chest tube side and bilateral PIFBs were placed at the end of surgery. Data regarding patient demographics, anesthesia method applied, amount of opioid used intraoperatively, cardiopulmonary bypass time, anesthesia and surgery time, postoperative extubation time, mechanical ventilation time, Visual Analogue Scale (VAS) of patients at rest and movement at 6th, 12th, 18th, 24th, 48th hours post-extubation, time to and type of first postoperative analgesic, postoperative complications, length of cardiac intensive care unit (CICU) stay and hospital length of stay were recorded from hospital records.

Results: The data of a total of 46 patients (Group 1: PECS II block + PIFB, n=20; Group 2: SAPB+ PIFB, n=26) were analyzed retrospectively. There was no difference in demographic variables between the groups. Intraoperative opioid usage, operation time, Cardiopulmonary bypass time, postoperative mechanical ventilation time, extubation time, ICU discharge time, and length of hospital stay were not statistically different between the groups. The first rescue analgesic requirement time was longer in group 2 than in group 1 but not statistically significant (18.76±15.36 h vs 12.62±10.61 h, p=0.162). The post-extubation VAS scores at rest and movement at the 6th hour were significantly lower in group 2 than in group 1 (1.73±1.28 vs 3.15±2.10, respectively, p=0.02).

Conclusion: In our study, the VAS scores at the 6th hour were lower in SAPB + PIFB group than in PECS II + PIFB group. As these blocks can be easy to apply, we thought these combinations could be an alternative for pain relief in cardiac surgery. Prospective randomized studies are needed with a large number of patients.

## Introduction

Cardiac surgery with surgical procedures such as sternotomy and mediastinal and thoracic tube insertion causes serious postoperative pain and discomfort [1]. In these patients, significant pain is seen in the postoperative period, and atelectasis of the lungs is frequently encountered because of inefficient respiratory effort due to this pain. It is reported that 33-75% of cardiac patients complained of moderate to severe postoperative pain despite treatment [2]. There is a risk of respiratory distress due to significant pain associated with cardiac surgery and consequently pulmonary morbidity. The adverse effects of inadequate pain management in the early postoperative period include pulmonary (atelectasis, decrease in functional residual capacity, pneumonia, and bronchial secretion stasis, etc.), cardiovascular (increased oxygen consumption, arrhythmia, tachycardia, etc.) and musculoskeletal system (diaphragm and intercostal muscles). Postoperative pain causes stress response and hyperglycemia. After extubation, patients who have difficulty coughing and moving due to pain may also have impaired oxygenation and a prolonged Intensive Care Unit (ICU) stay. On the contrary, effective postoperative analgesia in cardiac surgery aids early recovery and mobilization, provides early discharge from the ICU, and reduces hospital stay [3].

Multimodal analgesia treatment is recommended in cardiac surgery. The incidence of chronic pain after cardiac surgery was between 21% and 55% [4]. Therefore, adequate postoperative pain management is very important. In Enhanced Recovery After Surgery (ERAS) protocols, it was recommended that a multimodal opioid-sparing analgesic regimen for the treatment of postoperative pain for cardiac surgery patients. Also, an analgesic regimen should be designed to minimize the risk of delirium and facilitate early rehabilitation such as intravenous acetaminophen and a variety of regional anesthesia techniques [5]. A meta-analysis suggested that parasternal nerve block significantly reduced postoperative pain and opioid use [6]. Parasternal plane blocks might significantly improve outcomes for cardiac surgery with sternotomy and should be introduced comprehensively in ERAS protocols [7,8]. Regional analgesia is also a component of the optimal multimodal analgesia regimen [3]. As an alternative to central blocks (thoracic epidural), fascial plane blocks such as anterolateral chest wall (serratus anterior plane, pectoral nerve, pectointercostal nerve, etc.) and posterior chest wall (erector spinae plane nerve, paravertebral, etc.) have become more popular day by day [4]. Also, it is suggested that parasternal nerve blocks significantly reduce postoperative pain and opioid use [9]. It was mentioned that the Pecto-intercostal Fascial Block (PIFB) with ultrasound guidance can cover anterior branches of intercostal nerves from T2 to T6 [9]. The ultrasound-guided PIFB has been recommended for postoperative pain management in cardiac surgery [9,10]. Also, there were some doubts about the PIFB because it was mainly for median sternotomy-related pain and it would not cover the pain associated with chest drains and graft sites that happened in cardiac surgeries, although these were not as severe as sternotomy pain [11].

In our clinic, serratus anterior plane blocks (SAPB), pectoral nerve blocks II (PECS II), and PIFB are applied by ultrasonography (USG) as pain relief for both sternotomy and chest drain in the perioperative period to patients undergoing heart surgery. Our primary aim is to evaluate the severity of postoperative pain after chest wall blocks in patients who underwent open heart surgery. Our secondary aim is to evaluate the analgesic requirements time, extubation time, mortality, and morbidity of these patients, retrospectively.

## Materials and methods

After the approval of the ethics committee at our hospital (2011-KAEK-25 2022/06-15), 48 patients over the age of 18 years who underwent open heart surgery with sternotomy between September 1, 2021 and June 15, 2022 were evaluated retrospectively.

Patients who did not undergo chest wall block at the end of surgery were not included in the study. Patients with a history of cerebrovascular events, Alzheimer’s disease, dementia, inadequate cognitive functions, and emergency surgeries were excluded from the study.

After the patients were routinely monitored, general anesthesia was administered with intravenous midazolam 0.03mg/kg, thiopental sodium 5mg/kg, fentanyl 3-5mcg/kg, and rocuronium bromide 1mg/kg. The anesthesia was maintained with sevoflurane (MAC 1, 50% oxygen/air) and intravenous bolus doses of fentanyl (50mcg) and rocuronium bromide (10mg) intraoperatively. In Group 1 (n=20), the PECS II block was applied to the side of the chest tube. In Group 2 (n=26), SAPB was performed on the side of the chest tube. In both groups, PIFB was applied bilaterally 2cm lateral to the sternum at the end of the surgery. Following appropriate skin cleansing, a 21-gauge, 100 mm, Stimuplex® (B. Braun, Melsungen, Germany) block needle was inserted caudocranial or craniocaudal direction by using a linear high-frequency ultrasound transducer with in-plane technique After hydrodissection with normal saline to visualize the correct localization of the needle, 0.25% of bupivacaine was administered at a dose of 20ml or 0.2-0.4ml/kg. Totally 60ml of 0.25 % bupivacaine was given to all patients. No complications were observed during or after the block application. All patients were admitted to the cardiac intensive care unit (CICU) as intubated.

Demographic data, intraoperative amount of opioid, cardiopulmonary bypass time, anesthesia and surgery time, postoperative extubation time, mechanical ventilation time, Visual analog scale (VAS), postoperative first analgesic requirement time, type and amount of analgesic agent, postoperative complications (hypoxemia, hypercarbia, pneumothorax, pneumonia, reintubation), length of stay in cardiac intensive care unit and hospital, and mortality rate were recorded from the file of the patients. The severity of pain was classified as mild, moderate, and severe for analysis (mild=VAS 0-4, moderate=VAS 5-7, and severe=VAS >8). VAS scores were detected at six-time points: 0, 6, 12, 24, and 48 hours after extubation.

Statistical analysis

Statistical analysis was performed using the SPSS 22.0 (IBM Corp., Armonk, NY, USA) program. Descriptive statistics for numerical variables were expressed as mean ± standard deviation, while categorical data were expressed with numbers (n) and percentages (%). The compliance of numerical variables with normal distribution was evaluated with the Shapiro-Wilk test. The Mann-Whitney U test was used to compare those with non-normal distribution. Fisher's Exact test was used for categorical variables. The results were evaluated within the 95% confidence interval and p-values of 0.05 or lower were considered to show significance.

## Results

**Table 1:** Demographic data (n,%, mean ± SD) Manny Whitney U test, Fisher’s Exact test, LVEF: Left ventricular ejection fraction, BMI: Body Mass Index, *(F: Female, M: Male)

	Group 1 n=20	Group 2 n=26	P
Age (years)	58.15±11.85	57.42 ± 12.17	0.682
Gender F/M *	4/16 (20/80)	6/20 (23/77)	0.802
BMI	26.86±4.89	28.10±4.94	0.405
LVEF (%)	57.0±4.97	53.07±9.70	0.153
Euroscore II	3.20 ±1.85	2.84±1.37	0.625

In our study, 48 patients were enrolled, and two patients (one patient in Group 1 and one patient in group 2) were excluded from the study due to neurological disability in the postoperative period. Forty-six patients were divided into 2 groups (Group 1, n=20 and Group 2, n=26) retrospectively. There was no difference in demographic data between the groups (Table 1). Comorbidities, ASA status (American Society of Anesthesiologists classification status), and operation types (coronary artery bypass surgery, valve surgery, Bentall surgery) of patients were seen in Table 2.

**Table 2:** Comorbidities, ASA classification status, and operation types, n (%) DM: Diabetes Mellitus, HT: Hypertension, COPD: Chronic Obstructive pulmonary disease, Other: inflammatory disease, peripheric artery disease, MI: myocardial infarction in last 1 month, ASA: American Society of Anesthesiologists, CABG: Coronary artery bypass graft surgery

	Group 1 n=20		Group 2 n=26	
	n	%	n	%
Smoking status (Yes)	13	65	16	61.5
DM	6	30	7	26.9
HT	4	20	5	19.2
DM+HT	5	25	10	38.4
COPD	7	35	9	34.6
Other diseases	2	10	1	3.8
Preoperative MI	4	20	5	19.2
ASA 2	7	35	10	38
ASA 3	13	65	14	54
ASA 4	0	0	2	8
CABG	16	80	24	92.3
Valvular surgery	1	5	2	7.7
Bentall surgery	3	15	0	0

Intraoperative opioid use, operation time, cardiopulmonary bypass time, postoperative mechanical ventilation time, extubation time, discharge time from CICU were not statistically different between the groups (Table 3). First rescue analgesic requirement time was longer in Group 2 than in Group 1, but this difference was not statistically significant (18.76±15.36 and 12.62±10.61, respectively, p=0.162).

**Table 3:** Perioperative values (mean values ± SD) min: minute, h:hour, d:day, CICU: cardiac intensive care unit

	Group 1 n=20	Group 2 n=26	P
Operation time (h)	3.91±0.87	4.02±0.71	0.303
Cardiopulmonary bypass time (min)	94.0±19.84	102.5±34.30	0.542
Anesthesia time (h)	4.40±0.89	4,59±0,72	0.267
Intraoperative opioid use (µ)	875.0±168.19	959.61±247.79	0.118
Mechanical ventilation time (min)	336.50±101.63	316.15±93.77	0.510
Extubation time (min)	382.50±95.35	354.03±93.44	0.337
CICU discharge time (h)	55.70±3.93	82.34±120.50	0.849
First rescue analgesic requirement time (h)	12.62±10.61	18.76±15.36	0.162
Length of hospital stay (d)	6.25±2.73	7.11±5.43	0.855

VAS at rest and movement were evaluated at the 6th, 12th, 18th, 24th, and 48th hours after extubation. It was seen that VAS at the 6th hour was significantly lower in Group 2 than in Group 1 (1.73±1.28 and 3.15±2.10, respectively, p=0.02, Table 4).

**Table 4:** Postoperative Visual Analogue Scale Scores at rest and cough * p<0.05, Manny Whitney U test, VAS: Visual Analogue Scale, dVAS: Dynamic VAS(cough)

	Group 1 ( n =20)	Group 2 ( n=26)	P
VAS 0	1.15±2.15	0.53±1.52	0.362
VAS 6	3.15±2.10	1.73±1.28	0.024*
VAS 12	2.95±1.31	2.88± 1.36	0.758
VAS 24	3.25±1.44	3.53±1.74	0.579
VAS 48	1.20±0.89	1.92±1.69	0.207
dVAS 0	1.15±2.47	0.73±1.97	0.848
dVAS 6	4.30±1.94	2.69±1.40	0.005*
dVAS 12	4.15±1.30	3.92±1.35	0.438
dVAS 24	3.95±1.43	4.65±1.76	0.162
dVAS 48	2.25±1.11	2.96±1.77	0.236

In CICU, paracetamol 1000mg iv, tramadol 100mg iv, diclofenac 75mg iv and fentanyl 1µ/kg or combination of these were routinely used for rescue analgesia. The patient and surgeon satisfaction were evaluated as very satisfied, satisfied, hesitant, not satisfied (Table 5). 

**Table 5:** Rescue analgesics, patient and surgeon satisfaction, n (%) NSAIDs: Nonsteroidal anti-inflammatory drugs

	Group 1 (n=20)		Group 2 (n=26)	
	n	%	n	%
Paracetamol	13	65	13	50
Tramadol	2	10	1	3.8
NSAIDs	1	5	1	3.8
Paracetamol + NSAID	2	10	2	7.7
Fentanyl	1	5	0	0
Paracetamol + Tramadol	0	0	3	11.5
None	1	5	6	23.1
Patient Satisfaction				
Very Satisfied	4	20	15	57.7
Satisfied	7	35	4	15.4
Hesitant	6	30	6	23.1
Not Satisfied	3	15	1	3.8
Surgeon Satisfaction				
Very Satisfied	1	5	4	15.4
Satisfied	8	40	10	38.5
Hesitant	9	45	12	46.2
Not Satisfied	2	10	0	0

Postoperative complications in CICU as pneumonia, atelectasis, pneumothorax/subcutaneous emphysema, arrhythmia (atrial fibrillation, ventricular fibrillation, bradycardia), reintubation, high flow oxygen therapy or continuous positive airway pressure (CPAP) therapy after extubation, longed mechanical ventilation have been evaluated from intensive care follow-up forms (Table 6). Pneumonia and prolonged mechanical ventilation were seen in one patient in Group 2, and atelectasis was seen in five patients in Group 2 and four in Group 1. No mortality was seen in the first 30 days postoperatively in both groups.

**Table 6:** Postoperative complications in CICU, n(%) CICU: Cardiac intensive care unit, HFO: High flow nasal oxygen therapy, CPAP: Continuous Positive Airway Pressure

	Group 1 (n=20)		Group 2 (n=26)	
	n	%	n	%
Pneumonia	0	0	1	3.8
Atelectasis	4	20	5	19.2
Pneumothorax/subcutaneous emphysema	0	0	0	0
Atrial fibrillation	5	25	4	15.4
Reintubation	0	0	1	3.8
HFO	2	10	1	3.8
CPAP	0	0	2	7.7
Prolonged mechanical ventilation	0	0	1	3.8
Mortality	0	0	0	0

Since the blocks were performed by the same experienced anesthetists in our study, the application time of the blocks took approximately 20-25 minutes. Therefore, blocks were applied to patients who were hemodynamically stable at the end of the surgery. No complications were encountered and patients who could not be blocked by USG were excluded from the study.

## Discussion

We evaluated 48 patients who had open heart surgery between 01 September 2021 and 15 June 2022 retrospectively. Among VAS scores at rest and during movement, only the 6th-hour VAS scores in Group 1 (PECS II+PIFB) were significantly higher than in Group 2 (SAPB+PIFB).

Perioperative pain is both multifocal and multifactorial in cardiac surgery. Sternotomy, sternal retraction, internal mammary arterial dissection, posterior rib immobilization, and mediastinal and chest drains all contribute to pain experienced in the acute postoperative period. Ineffective pain management can result in systemic and pulmonary complications with significant cardiac consequences. Postoperative pain in cardiac surgery is originated from surgical manipulation and trauma. Surgical incision, retraction, and suturing cause an inflammatory response, and acute pain occurs [12]. In the early postoperative period, pain is worst and generally decreases with tissue healing. Risk factors for severe postoperative pain after cardiac surgery are younger patient age (< 60 years old), the type of surgery, and longer duration of surgery [4]. We preferred to apply fascial plane blocks because the mean age of our patients was under 60 years and our surgical time was relatively longer (3.91±0.87 hours in Group 1, 4.02±0.71 hours in Group 2).

Kaya et al. investigated the efficiency of bilateral PIFB or transversus thoracic muscle plane block (TTMPB) on acute poststernotomy pain in 39 cardiac surgery patients and concluded that the first 24-hour morphine use did not significantly differ between the PIFB and TTMPB groups [13]. They reported that PIFB and TTMPB showed similar effectiveness in acute poststernotomy pain scores. Also, we applied combined fascial plane blocks (PECS II+PIFB and SAPB+PIFB) at the end of the surgery. The first analgesic requirement time was longer in Group 2 but not statistically significant and VAS scores at the 6th hour were statistically significantly lower in Group 2 than in Group 1. We think that this is the discomfort and pain caused by the thoracic tube applied to the patients, and this provides more comfort with SAPB. Therefore, patient satisfaction in our study is higher in Group 2 (57%) than in Group 1 (20%).

In a prospective study, Kumar et al. investigated the efficacy of bilateral pectoralis nerve block (PECS I+II) for ultrafast tracking and postoperative pain management in cardiac surgery [14]. The authors added dexmedetomidine 25μg as an additive to 0.25% bupivacaine and totally a volume of 30 ml of local anesthetic solution was given bilaterally. In the PECS block group, pain scores at rest were significantly lower in patients at 0, 3, 6, 12, and 18 h after extubation than in the control group (P<0.05). There was no statistically significant difference found at the 24th h VAS scores (rest and cough) between the two groups (P=0.6832). They mentioned that bilateral PECS block reduced VAS scores postoperatively [14]. We applied PİFB bilaterally and PECS II block to the chest tube side. Also, for each block, a volume of 20 ml of local anesthetic solutions without an additive agent was given.

There were many studies with SAPB for analgesia in minimally invasive heart surgery [15-17]. Berthoud et al. [15] mentioned that VAS scores were significantly lower in the SAPB group compared to continuous wound infiltration (p < 0.01). In a meta-analysis of randomized trials with SAPB for cardiothoracic surgery and trauma, authors concluded that the use of SAPB was safe and effective [17]. In our study, we applied SAPB together with PIFB for postoperative pain relief in Group 2. While VAS scores were found to be low in the first 12 hours after extubation in this group, the VAS score was significantly lower only at the 6th hour compared to PECS II together with PIFB. Although there was no significant difference between the groups, the first analgesic time was longer and the extubation time was shorter because of adequate analgesia in Group 2.

In another study, the efficacy of Ultrasound-Guided SAPB, PECS II Block, and intercostal nerve block (ICNB) was compared after pediatric cardiac surgery. They found that SAPB and PECS II blocks were equally efficacious in post-thoracotomy pain management compared with ICNB and these blocks were easily applied as the traditional ICNB [16]. We think that ultrasound-guided SAPB and PIFB can be easily applied in adult patients, and they are effective for pain relief in cardiac surgery.

In a retrospective study, the authors performed many types of thoracic fascial plane blocks (thoracic paravertebral block, double injection erector spinae plane block-ESP, SAPB, parasternal intercostal block, or combination of blocks) on patients who had cardiac surgery preoperatively. The facial plane block was applied to 179 patients out of 220 patients in five years period. Opioid consumption in the first 24h postoperatively, length of stay in CICU, and first mobilization time were decreased in facial plane block groups. They reported that the most opioid consumption was in the SAPB + parasternal intercostal block group which included only five cases. But they applied these blocks before the beginning of surgery [3]. In another prospective study, ESP and a combination of ESP and superficial parasternal intercostal plane (S-PIP) blocks were applied for post-sternotomy pain at the beginning of cardiac surgery. The combination of ESP and S-PIP blocks lowered pain scores and postoperative morphine requirement [18]. In this study, anesthesia techniques, surgery time, and intraoperative opioid consumption were similar to our study. In our study, the blocks were preferred to apply at the end of the surgery to prolong the effectiveness of postoperative analgesia.

All patients were operated on with cardiopulmonary bypass and the duration of surgery was longer in our study. Intermittent bolus doses of fentanyl were used intraoperatively for cardiac surgery in our hospital and the intraoperative total amount of opioids was higher than in other studies [3,14,18]. Due to fascial plane blocks and a higher amount of intraoperative opioid use, the need for rescue analgesia with opioids was lower postoperatively.

We searched for postoperative complications of patients. They were similar between the groups. Prolonged extubation time (>24 hours) and prolonged mechanical ventilation are related to the increased risk of pneumonia [19]. Postoperative pneumonia occurrence is reported at 9.96% in adult patients after cardiac surgery [20]. Pneumonia was in 3.8% of patients in group 2 in our study.

Intravenous acetaminophen has been widely used for analgesia and opioid consumption reduction has been seen in cardiac surgery [21,22]. In our study, it was seen that paracetamol 1000mg intravenously was mostly used for analgesia. Secondly, tramadol and nonsteroidal anti-inflammatory drugs were used for analgesia.

In our study, patients’ mean body mass index was ≥ 25 in both groups. It was reported that overweight or obese (body mass index≥ 25) patients had higher pain intensity than normal-weight patients on postoperative days in cardiac surgery, and obese patients had greater pain sensitivity [23,24]. We think that the fascial plane blocks application in these patients and the absence of postoperative complications for this cause an increase in surgeon and patient satisfaction.

Study limitations

There are some limitations in our study. This study has a retrospective nature. The single-center structure of the study, the small number of patients, the short period of screening, and the absence of a control group are placed in our limitations. Due to the absence of a control group, further statistical analysis could not be performed. The dermatomal analysis could not be performed in both groups because the blocks were performed under general anesthesia at the end of surgery. Since pain monitoring of patients could not be performed intraoperatively, opioids were administered with a standard protocol in both groups in our study. 

## Conclusions

The importance of facial plane blocks, one of the postoperative analgesia techniques, that are significantly effective in terms of rapid recovery criteria in cardiac surgery is increasing day by day. According to our study, SABP+PIFB was the facial plane block that provided more effective analgesia in the early postoperative period than PECS+PIFB in cardiac surgery. Therefore, SAPB with PIFB could be a good alternative to other fascial plane blocks for pain relief after cardiac surgery. Since these block combinations can be easily performed with USG, we think that they should be included more as a part of multimodal pain management in cardiac surgery. More comprehensive, randomized, controlled further studies are needed to determine the efficacy and safety of different regional techniques for pain control after cardiac surgery.
